# Performance Evaluation of Container Orchestration Tools in Edge Computing Environments

**DOI:** 10.3390/s23084008

**Published:** 2023-04-15

**Authors:** Ivan Čilić, Petar Krivić, Ivana Podnar Žarko, Mario Kušek

**Affiliations:** Faculty of Electrical Engineering and Computing, University of Zagreb, 10000 Zagreb, Croatia

**Keywords:** edge computing, service scheduling, service orchestration, container, Internet of Things (IoT), Kubernetes, K3s, KubeEdge, ioFog

## Abstract

Edge computing is a viable approach to improve service delivery and performance parameters by extending the cloud with resources placed closer to a given service environment. Numerous research papers in the literature have already identified the key benefits of this architectural approach. However, most results are based on simulations performed in closed network environments. This paper aims to analyze the existing implementations of processing environments containing edge resources, taking into account the targeted quality of service (QoS) parameters and the utilized orchestration platforms. Based on this analysis, the most popular edge orchestration platforms are evaluated in terms of their workflow that allows the inclusion of remote devices in the processing environment and their ability to adapt the logic of the scheduling algorithms to improve the targeted QoS attributes. The experimental results compare the performance of the platforms and show the current state of their readiness for edge computing in real network and execution environments. These findings suggest that Kubernetes and its distributions have the potential to provide effective scheduling across the resources on the network’s edge. However, some challenges still have to be addressed to completely adapt these tools for such a dynamic and distributed execution environment as edge computing implies.

## 1. Introduction

Most of today’s Internet of Things (IoT) data traffic is transmitted over the Internet towards remote cloud servers for processing or storage. However, such an architectural approach gradually leads to a buildup of network congestion and results in a prolonged overall processing cycle for IoT services and reduced responsiveness to events detected in local smart environments. The concept of Edge-to-Cloud Continuum (ECC) emerged to reverse this trend and significantly reduce the traffic generated towards the cloud by enabling the processing of IoT data closer to the data sources.

**Figure 1:**
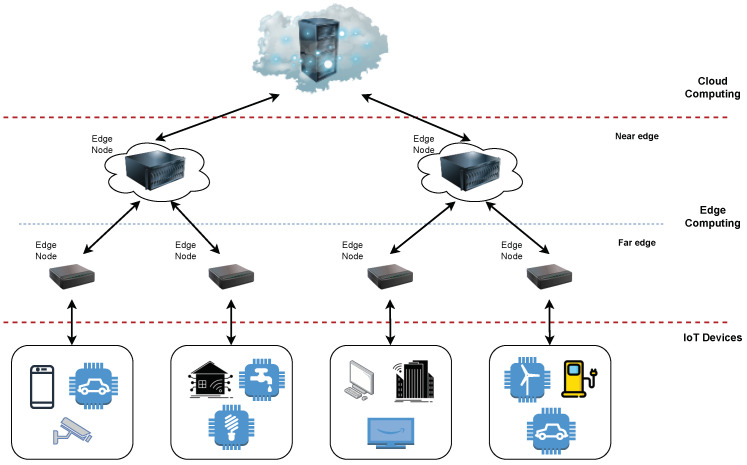
Abstract view of the Edge-to-Cloud continuum.

In the ECC, devices are organized hierarchically into layers, as shown in Figure 1. A more detailed description of entities in each layer of the depicted architecture is given in our previous work [1]. It is important to note that each layer offloads the upper layer and executes a certain amount of its functionalities. Additionally, nodes within the same layer are also mutually connected to share the processing load and optimize the placement of the deployed services. Thus, the processing and storage capabilities are brought closer to the end devices, which offers the opportunity to achieve the following critical goals of IoT concept [2]: reduced overall network traffic, improved responsiveness and shorter processing cycle, enhanced security with privacy control, and lower operational costs.

Since the emergence of the ECC concept, the idea of extending the cloud with computing resources placed closer to IoT devices and end-users has been extensively covered in the academic literature. The aforementioned benefits of ECC, introduced by the additional edge layers, are well known: In theory, efficient utilization of ECC resources should improve the performance of IoT services by executing processing closer to data sources and service consumers. However, adding heterogeneous computing resources at the edge of the network and service orchestration in such a distributed and dynamic execution environment poses a significant challenge in practice. In such an execution environment, where end devices and edge nodes are constantly changing their state and location, manual service management becomes complex and should be avoided by utilizing the automated approach enabled by an appropriate orchestration tool, e.g., Kubernetes, KubeEdge, K3s, or ioFog. Thus, services running on the edge nodes should be automatically orchestrated to maintain their high availability.

In addition, an edge service assigned to manage or process data from an IoT device must be autonomous, stateless, and portable to ensure that edge service migrations across the ECC are short, while service availability remains high. The best solution for easy-to-migrate services is container virtualization because, once packaged, a service can easily be migrated with reduced startup time compared to the other methods. Thus, efficient service orchestration combined with portable containerized components of IoT services is required to achieve ECC benefits in practice. For that reason, throughout this paper, we will focus specifically on container orchestration tools. These tools were primarily designed to manage the deployment of containerized applications in large-scale clusters and are capable of running hundreds of thousands of jobs across thousands of machines [3], and in this paper, we will analyze how they can be applied to a distributed and dynamic ECC environment.

Service orchestration in the ECC implies scheduling, deploying and managing services based on a specific scheduling policy within a dynamic and unstable execution environment. The challenge of implementing efficient service orchestration has been analyzed mainly in the scope of cloud administration before the emergence of edge computing. Thus, different approaches and orchestration systems already exist, but they must be adapted to become more suitable for the edge computing environment. Current implementations of orchestration tools typically involve many features and capabilities necessary to ensure system scalability and reliability across cloud environments. However, such capabilities make them resource-demanding and often too heavy for the devices at the network’s edge. Furthermore, as these numerous features are not critical in edge computing use cases, their number could be reduced to achieve optimized and lightweight versions of existing orchestration tools. Such versions should primarily include features necessary to execute efficient orchestration in the edge computing environment, where the emphasis should be on specific performance targets rather than goals critical for cloud-based systems.

Although such goals, critical for orchestration in the cloud environment, are not excluded, service orchestration in edge computing should be pointed more towards utilizing the available computing resources to ensure the desired QoS and improved overall system performance. Such a goal presents a challenge as the QoS target differs depending on the goal of a specific use-case scenario. Thus, different use cases should apply custom scheduling policies to properly exploit the benefits of the edge computing concept in a specific scenario and reach the targeted QoS level. Therefore, an important requirement for service orchestration platforms that are to be used within the ECC is to enable the adjustments of scheduling policies that determine the execution logic of service orchestration. Another differentiating factor between service orchestration in a cloud environment and edge computing is that the involved computing edge nodes are placed across distant and separate local area networks, often without static public IP addresses. This poses another challenge for the adaptation of the existing orchestration tools since the orchestrating node has to be able to issue commands towards entities without public IP addresses and outside of its own private network, which is usually not the case within cloud environments.

The two aforementioned requirements, namely, custom scheduling policies and ease of configuration across different networks, are the focus of our examination of the existing tools for container orchestration within edge computing environments. To obtain relevant inputs for our comparison of orchestration tools, we first conducted a survey of state of the art in service scheduling and orchestration at the edge and categorized the works based on the predefined parameters. The main objective of the survey is to analyze how often orchestration tools were used to evaluate the proposed approaches and which are the most commonly used tools. Furthermore, if the evaluation was done by simulation, we wanted to identify the main reason for avoiding evaluation in a real environment and utilizing an existing orchestration tool. Therefore, as input to our experiments on tool evaluation, the survey had to provide the following answers: (i) what are the most common QoS parameters that were considered for the implementation of custom service scheduling; (ii) what is the most common hardware used to run the edge nodes. Based on this information and the two requirements mentioned above, we then analyze the complexity of utilizing the existing orchestration tools for the implementation of custom service scheduling in a common edge execution environment.

Our main motivation for writing this paper stems from the need to provide a summary and evaluation of the main container orchestration tools for the edge domain to facilitate the selection of an appropriate container orchestration tool for edge computing solutions. Therefore, we summarize the current state of the art in edge orchestration to classify the work according to the scheduling strategies and the use of container orchestration to provide input for the evaluation of container orchestration tools and highlight current trends in edge orchestration.

A relevant survey focusing on containerization and scheduling of edge services is reported in [4]. The authors provide an overview of Kubernetes and Docker Swarm schedulers, as well as algorithms used to efficiently schedule container-based services in edge computing environments. In comparison, our work examines a larger set of edge-oriented container orchestration tools (K3s, KubeEdge, and ioFog) and provides their comparative performance evaluation within a real-world edge deployment. The authors in [5] also provide a survey of edge orchestration, focusing on container orchestration tools. They analyze similar tools (Kubernetes, KubeEdge, K3s, and ioFog) with an emphasis on theoretical evaluation, while we provide a practical deployment example and performance evaluation of these tools in a real-world environment. A similar study is reported in [6]. The work provides an evaluation of the performance impact of using Docker containers for IoT applications in fog computing infrastructures. Finally, the authors propose a framework for running IoT applications at the edge based on Docker Swarm. Compared to our experiments, they measure container overhead, while we measure the overhead of existing container orchestration tools once our service is containerized. A thorough survey of fog/edge orchestration challenges was conducted in [7]. The authors provide an overview of state of the art in service orchestration and present technologies that can be used to overcome the major orchestration challenges. Compared to their overview of related work, we focus more on orchestration tools, while their work focuses mainly on edge-enabling technologies such as NFV, SDN, or serverless computing, and the challenges of implementing these technologies.

Our contribution can be summarized as follows:

- We provide an overview of state of the art in edge orchestration, focusing on how orchestration has been implemented and what hardware has been most commonly used.
- We provide an architectural analysis of selected container orchestration tools, i.e., Kubernetes, K3s, KubeEdge and ioFog, focusing on their readiness for use in a resource-constrained edge environment and the possibilities for implementing custom service scheduling algorithms.
- We evaluate the selected container orchestration tools in an experiment to investigate how they perform in a real edge computing environment regarding memory footprint on a resource-constrained edge node and their support for edge nodes in private networks. Finally, we provide a performance evaluation to determine the startup and migration time of our custom edge service.

The work is organized as follows. Section 2 gives an overview of related work in the field of ECC orchestration. Section 3 gives an architectural overview of selected container orchestration tools with respect to their applicability in the edge domain. Section 4 provides a comparison of selected container orchestration tools based on cluster deployment and performance evaluation. Finally, Section 5 provides the conclusion and lists future work.

*Short note on terminology*. The term *fog computing* is frequently used in the literature, and in fact both edge and fog computing refer to the same technology: a distributed computing architecture that brings cloud services closer to end devices in the edge-to-cloud continuum. In some related works, fog and edge computing are divided into different layers, where the edge layer represents the far edge, placed at the first network hop from the end devices, while the fog layer is placed between the edge and the cloud layer (near edge). In this paper, we prefer to use the term *edge* as it has been used more frequently in the scientific literature recently.

## 2. Extensive Survey of Relevant Works in the Area of Edge Orchestration

The paper aims to compare existing container orchestration tools in terms of their applicability for edge computing implementations in the IoT domain. Our main motivation for this comparison stems from the fact that academic works in the field of edge computing often perform simulations without performing an evaluation in a real-world environment using service orchestration tools. Therefore, we survey related work to identify works that employ service scheduling, optimized service placement, or service orchestration in IoT-based edge environments regardless of their evaluation methodology. Based on this review, we aim to answer the following research questions and use it as the basis for our performance evaluation of orchestration tools:

1. What type of service scheduling (static or dynamic) is mainly utilized and what QoS parameters are used for scheduling?
2. Are orchestration tools used for deploying and managing services at the edge, and if not, what are the main reasons for employing simulations to evaluate the proposed algorithm/solution?
3. What is the main type of hardware being considered for running far- and near-edge nodes?

Table 1 shows queries used in the search for relevant works in IoT service orchestration at the edge. The search was performed in the Web of Science Core Collection [8] database. The table shows how many relevant papers were obtained with the given queries, and the results are not distinct, i.e., the same paper can be obtained with multiple queries. It is important to emphasize that not all results of the queries were considered, but results were manually filtered to obtain only relevant works that employ service scheduling, optimized service placement, or service orchestration in IoT-based edge environments. Both terms *edge* and *fog* were used as keywords in the queries as it is still not standardized in the literature. The table shows that the most relevant works were found when the terms *orchestration* and *container* were used as keywords, which makes sense since container orchestration is our main focus in this work.

The problem of service orchestration in edge computing environments is a well-known research topic. Table 2 gives an overview of related work in the field of edge service orchestration with the five main categorization questions:

- *ECC layer*: Which ECC layer from the Figure 1 was targeted within the paper?
- *Scheduling*: Was service scheduling used? Was it static or dynamic scheduling?
- *QoS parameters*: If service scheduling was used, what QoS parameters were used for scheduling? Three parameters were considered for this classification: response latency, request throughput and privacy requirements.
- *Containers*: Were containers used for service deployment?
- *Orchestration tool*: What orchestration tool was used for service deployment and management? Are the authors using general-purpose container orchestration tools, such as Kubernetes [9] and Docker Swarm [10], container orchestration tools designed for the edge, such as K3s [11], KubeEdge [12], or ioFog [13], or if the services are running as VMs, are they using tools such as OpenStack [14] to manage this virtualized environment?
- *Edge node hardware*: What hardware was used for the edge node implementation? If the edge environment was simulated or no experiment was performed, no hardware information is provided.

**Table 2:** Related work in the field of edge service orchestration.

Reference	ECC Layer	Scheduling	QoS Parameters	Containers	Orchestration Tool	Edge Node Hardware
[15]	NE	Dynamic	L, T	Yes	- (custom)	Not specified
[16]	NE	Dynamic	L	Yes	ioFog	VM: 2 vCPU, 2 GB RAM
[17]	FE, NE	Dynamic	L, T	Yes	- (simulated)	-
[18]	FE	Dynamic	T	No	- (simulated)	-
[19]	FE, C	Dynamic	L	No	- (simulated)	-
[20]	NE	Dynamic	L	No	- (simulated)	-
[21]	FE, C	Dynamic	L	Yes	Docker Swarm	VM: 1 vCPU, 1 GB RAM
[22]	NE, C	Dynamic	L	No	-	Raspberry Pi 3
[23]	FE	Dynamic	L	Yes	OpenWhisk	Intel Xeon CPU
[24]	FE, NE, C	Dynamic	L, T	Yes	- (custom)	Raspberry Pi 3
[25]	FE, NE	Static	L	Yes	- (custom)	Raspberry Pi 3
[26]	FE	-	-	Yes	Docker Swarm	-
[27]	FE, NE, C	Dynamic	L	No	-	VM: 1 vCPU, 1 GB RAM
[28]	FE, NE, C	Dynamic	L, T, P	Yes	Kubernetes	Raspberry Pi 3
[29]	NE, C	Dynamic	L	No	- (simulated)	-
[30]	FE	-	-	Yes	OpenWhisk	12 CPU cores, 16GB RAM
[31]	FE	-	-	No (WebAssembly)	- (custom)	Intel Xeon E5-2680 v2 (CPU)
[32]	FE, C	Dynamic	L, T	No	Openwhisk	VM: 2 or 4 vCPU, 4 GB RAM
[33]	FE	Dynamic	L	No	-	Not specified
[34]	FE, C	Dynamic	L	No	-	VM
[35]	FE, C	Dynamic	L, T	No	- (simulated)	-
[36]	FE, C	Dynamic	L	No	- (simulated)	-
[37]	FE	Static	-	Yes	Kubernetes	Laptop Asus x507ma; VM: 4 vCPU, 3 GB RAM
[38]	FE, C	Dynamic	L, T	Yes	K3s	Not specified
[39]	FE, C	Dynamic	-	Yes	Kubernetes, KubeEdge	ARM64 4 core CPU, 4 GB RAM
[40]	FE, C	Dynamic	L, T, P	Yes	- (custom)	-
[41]	NE	Dynamic	L	No (VMs)	OpenStack	Dell PowerEdge R530
[42]	FE, NE, C	Dynamic	L, T, P	No	CometCloud	Raspberry Pi
[43]	FE, C	Dynamic	L	No	- (simulated)	-
[44]	FE, C	Dynamic	L, T	Yes	KubeEdge	Jetson AGX Xavier; Jetson Nano; Raspberry Pi 3
[45]	NE, FE, C	Dynamic	L, T	Yes	Kubernetes	Raspberry Pi 3; VM: 2 vCPU, 7.5 GB RAM
[46]	FE, C	Dynamic	L	No (VMs)	- (custom)	Dell PowerEdge R530; Intel NUC; Dell Optiplex 7050
[47]	FE	Dynamic	L, T	Yes	- (custom)	Desktop with Intel i7-6700K CPU
[48]	NE, C	-	-	Yes	Kubernetes	Intel Xeon E5-2609 (CPU); Intel Core i7-6700 CPU
[49]	FE	Dynamic	L, T, P	Yes	- (custom)	4 CPU cores, 16 GB RAM
[50]	NE, C	Dynamic	L	Yes	- (custom)	Not specified
[51]	FE	Dynamic	L, T	Yes	- (simulated)	-
[52]	FE, NE	Dynamic	L	Yes	- (custom)	Raspberry Pi 3; Intel Core i5, 8GB RAM
[53]	FE, NE, C	-	-	Yes	K3s	VM: 2vCPU, 4 GB RAM
[54]	FE, NE, C	Dynamic	L, T	Yes	Kubernetes	Raspberry Pi; Jetson TX2; Intel NUC
[55]	NE, C	Dynamic	L, T	Yes	- (simulated)	-
[56]	FE	-	-	Yes	-	Raspberry Pi 3; Dell PowerEdge C6220
[57]	NE	Dynamic	L	Yes	- (simulated)	-
[58]	FE	-	-	Yes	- (custom)	Raspberry Pi 3; Laptop ASUS Zenbook UX331UN
[59]	NE, C	-	-	Yes	Docker Swarm	Intel Core i3-2120 CPU, 8 GB RAM
[60]	NE, C	Dynamic	-	Yes	- (custom)	Odroid C2 (ARM Cortex-A53, 2 GB RAM)
[61]	NE	Dynamic	L	No	- (simulated)	-
[62]	FE, NE	Dynamic	L	No	- (simulated)	-
[63]	FE, NE, C	Dynamic	L, T	No	- (simulated)	-
[64]	FE, NE, C	Dynamic	L	Yes	- (simulated)	-
[65]	FE, NE, C	Dynamic	L	Yes	Kubernetes	Raspberry Pi
[66]	FE	Dynamic	-	Yes	Kubernetes	Jetson TX1, TX2, Nano, Xavier; Raspberry Pi with Google Edge TPU
[67]	FE, NE	Dynamic	L, T	Yes	Kubernetes	Not specified
[68]	FE, NE	Dynamic	L, T	Yes	KubeEdge	4 CPU cores, 4 GB RAM
[69]	NE	Dynamic	L	No	- (simulated)	Jetson Nano

FE—Far Edge; NE—Near Edge; C—Cloud | L—Latency; T—Throughput; P—Privacy.

**Figure 2:**
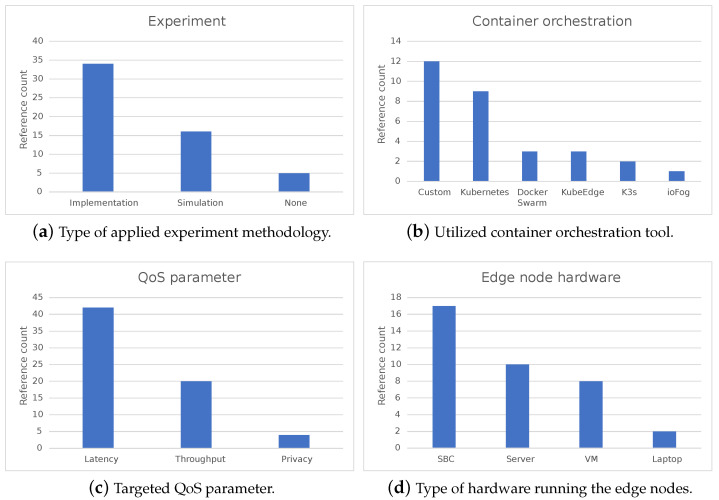
Summary of a related work survey (distribution by category).

Figure 2 is the visual representation of the results from Table 2. Figure 2a provides the classification of works according to the experiment performed, while Figure 2b classifies works that use container orchestration according to the tool used or if a custom implementation was provided. Figure 2c indicates the number of works that utilize a particular QoS parameter for service scheduling, and Figure 2d shows the hardware used to run edge nodes with the number of occurrences in the corresponding works.

The main conclusion from the provided state-of-the-art overview is that container orchestration tools are not yet widely utilized in edge orchestration works, as can be seen in Figure 2a,b. One of the main reasons for this is that simulation (which is still a popular validation technique) is more convenient for solution evaluation when a large number of edge nodes need to be simulated along with an underlying network. For example, some works require simulations of more than 1000 devices to validate the scalability of the system, which is reasonably difficult and expensive to implement to prove the value of the proposed algorithm, e.g., [35,62,63]. The following quote summarizes the problem faced by the authors in evaluating edge-oriented algorithms: “*Running comprehensive empirical analysis for the resource management algorithms in such a problem would be very costly, therefore, we rely on simulation environment*” [62]. Another problem the authors are facing is the implementation of a MEC (Multi-access Edge Computing) environment [70]. MEC offers cloud computing capabilities at the edge of telecom operators’ infrastructure, which makes it difficult to conduct experiments given the lack of testbeds. Therefore, we believe that the authors of MEC-based works are more keen on providing evaluations through simulations, as noted in [18,35,55,69]. Finally, we must point out that existing container orchestration tools: (i) bring deployment complexity; (ii) make it difficult to implement custom scheduling algorithms, and (iii) are mainly not designed for resource-constrained edge environments. We concluded this due to the fact that most authors implement their own orchestration solution or use Kubernetes [45,65,66,67]—which is not designed for edge environments—or Docker Swarm [21,26,59]—which is no longer being developed nor supported.

However, the only way to determine the overhead incurred by orchestration, e.g., when deploying a service on a resource-constrained edge node and migrating the service to a new node, is to perform an experimental evaluation in an operational environment. Moreover, a resource-constrained environment needs to be continuously monitored to evaluate the performance of a scheduling algorithm in practice. In addition, simulation tools are not able to simulate with high precision the underlying network, which may be unstable at the edge, as well as the hardware heterogeneity, which significantly affects the performance of services at the edge. Therefore, we will provide the evaluation of existing container orchestration tools in terms of their applicability for edge service orchestration and scheduling on resource-constrained edge nodes.

Figure 2c shows that most of the works implementing scheduling use latency as the main scheduling parameter. The main reason for this is that reducing latency is the most emphasized benefit of edge computing, while preserving bandwidth and privacy is also important, but not as prominent in state of the art. Moreover, developing latency-aware scheduling algorithms is much more straightforward than bandwidth- or privacy-aware scheduling. Finally, Figure 2d shows that most of the experiments performed on real hardware are implemented on single-board computers (SBCs). This will serve as input for our evaluation of container orchestration tools since one of the requirements is that the tool can run on resource-constrained SBCs. In addition, it can be noted that the most common VM setup for the edge node is up to 2 vCPU and 2 GB RAM.

## 3. Overview of Selected Container Orchestration Tools

Based on the survey reported in Section 2, we selected the following four orchestration tools for our evaluation of their applicability within the edge computing environment: Kubernetes, K3s, KubeEdge, and ioFog. We did not consider Docker Swarm as it is not intended for managing distributed workloads, and it is often introduced as a simplified alternative to Kubernetes, which does not offer its advanced automation features and high customization. Our choice was primarily determined by the frequency of their utilization in relevant papers analyzed in Section 2. In addition, we also considered tools that are utilized less frequently but are designated specifically for edge environments. Most of these tools (such as K3s and KubeEdge) are built upon Kubernetes but have been simplified to provide a lightweight alternative that is more suitable for resource-constrained nodes. In contrast, ioFog is a standalone orchestration tool that is built on its own architectural principles.

### 3.1. Architectural Analysis of Selected Orchestration Tools

Before providing an overview of the four orchestration tools we have selected, we analyze their architectural components to gain a better understanding of their operational logic. The description of each tool provides insight into its operational logic, so this section offers the opportunity to better understand their differences before the experimental evaluation of their performance.

#### 3.1.1. Kubernetes

Kubernetes is widely recognized as the preeminent open-source platform for container orchestration. As defined in [9], Kubernetes is an open-source system designed to automate the deployment, scaling, and management of containerized applications. It offers a wide range of capabilities such as configuration management, self-recovery, automated rollouts and rollbacks, and load balancing that together make it probably the most popular orchestration tool today.

Kubernetes enables the creation of a computer cluster composed of a group of operational machines known as nodes, which are responsible for executing containerized applications. Worker nodes serve as hosts for the execution of application components which in Kubernetes terms are referred to as pods. The control plane, which may be deployed on a single node or across multiple nodes, oversees the workers and pods within the cluster. Its key responsibilities include making high-level decisions about the cluster and responding to the detected events that occur within the cluster.

**Figure 3:**
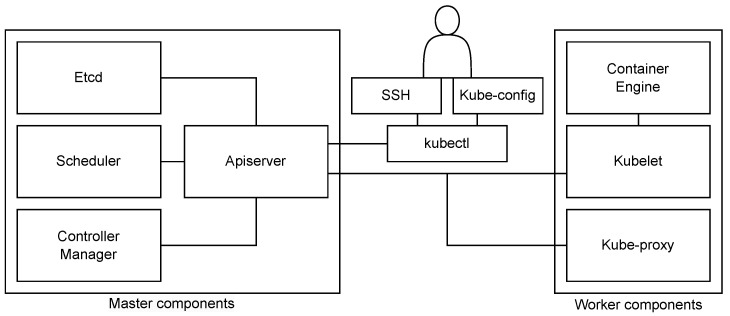
Kubernetes architecture.

The control plane consists of several components: kube-apiserver, etcd, kube-scheduler, kube-controller-manager, and cloud-controller-manager (Figure 3). These components can be run on any machine in the cluster, and together, they enable efficient cluster management. Worker nodes of the cluster run the following node components: kubelet, kube-proxy, and container runtime. They are used to run pods and thus provide the Kubernetes runtime environment [71].

As Kubernetes originally was not intended for deployment across distributed locations, it is not applicable for service orchestration within an edge computing environment in its current form. However, with certain adjustments, it can be adapted to support remote worker nodes and offer the possibility of implementing a functional orchestrated edge layer. The main obstacle towards including remote worker nodes in a Kubernetes cluster is the prerequisite of continuous communication between the control plane and worker nodes. The communication from worker nodes towards the control plane terminates at the exposed interface of the API server component [72]. Remote worker nodes are able to communicate with the control plane exposing public IP addresses since, in the edge computing environments, the control plane is expected to run on a publicly available cloud infrastructure due to its resource-intensive components [73]. However, the control plane should have two primary communication paths towards the included worker nodes: one towards the kubelet that is terminated at the kubelet’s HTTPS endpoint (for fetching pod logs, attaching to running pods, and providing the kubelet’s port-forwarding functionality), and the second towards nodes, pods and services. In a cluster environment, such communication is enabled since worker nodes are within the same network environment or they at least have available IP addresses. However, in the desired edge computing model, where the worker devices could be placed in remote private networks without static public IP addresses, this becomes a challenge that has to be resolved.

Other drawbacks of utilizing Kubernetes for edge orchestration are consequent to its strong reliance on high network and processing performance that is available within the cloud environments, while the edge usually implies reduced resource abilities. This is why today there are multiple orchestration tools that are built upon Kubernetes or that utilize a similar approach but offer service orchestration that is more suitable for the edge computing environment.

The motivation for building an edge computing platform based on Kubernetes was to provide a solution that would enable the inclusion of remote and constrained devices in the processing plane. The main challenges for the successful implementation of such a platform are limited resources at the edge, unstable network environments, possible network outages, and device heterogeneity. Thus, the platform has to extend Kubernetes functionality, but it also has to be lightweight, enable interaction with devices in private networks with lower bandwidth, support autonomy at the edge (offline mode) and support various devices and protocols. Also, multiple Kubernetes distributions that specifically target edge environments are available today, which confirms the premise of its inapplicability for its straightforward utilization in architectures that include the edge processing layers.

#### 3.1.2. K3s

K3s is a Kubernetes distribution designed to distribute processing workloads across remote environments utilizing the available constrained edge devices. As described in its official documentation [11], K3s is a lightweight Kubernetes version with a binary of less than 100 MB in size that supports ARM, which makes it a good candidate for the utilization in edge computing with constrained devices. Its binary contains all Kubernetes control plane components encapsulated within a single process, while the number of external dependencies is minimized.

K3s architecture also consists of two parts: server node running the *k3s server* command and worker node running the *k3s agent* command (components booted by these commands are the ones already described in Figure 3). Agent node connects to the server node establishing a WebSocket connection which is then maintained by the load balancer running as a part of the agent process. Depending on a specific use case scenario, two different server setups can be established: a simple single-server setup with an embedded database and a highly available setup that implies multiple server nodes with an external database. A worker establishes a connection with the server using the server’s unique token and its URL, or a fixed registration address in case of a highly available setup. After registration, the agent node establishes a connection directly to one of the server nodes.

K3s does not differ much from Kubernetes, but due to its lightweight binary and ARM chip support, it should be more suitable for edge computing use cases. Another advantage that makes K3s a more appropriate solution than Kubernetes for the edge computing environment is its ability to include devices without public IP addresses. This is achieved by setting up a tunnel proxy on a device that runs the K3s agent. Once the K3s agent is up and running, a tunnel proxy will be installed to establish a connection with the controller. The data traffic between the agent node and the controller can then be exchanged securely through this newly established bidirectional link [74].

#### 3.1.3. KubeEdge

KubeEdge is an open-source platform designed to enhance container orchestration and cluster management at the network’s edge [12]. It leverages the well-established Kubernetes framework to offer infrastructural support for networking, application deployment, and metadata synchronization between the cloud and resources at the network’s edge. Also, the platform additionally supports the MQTT protocol and empowers the implementation of customized logic to offer a mechanism that facilitates communication with devices at the network’s edge.

**Figure 4:**
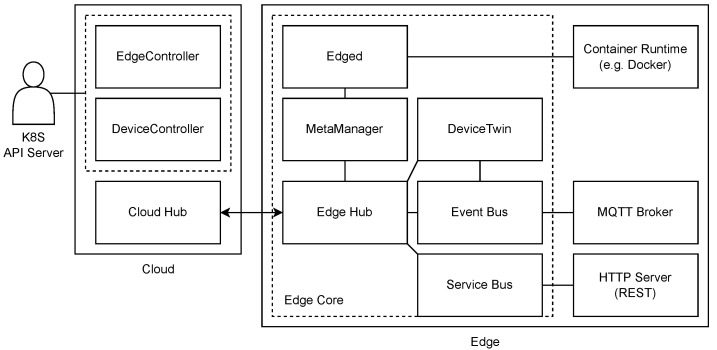
KubeEdge architecture.

The architecture of KubeEdge consists of two separate parts: cloud and edge (Figure 4). Each part contains multiple components that together provide support for extending the cloud with edge resources. The cloud part that typically resides within the cloud environment includes three modules: EdgeController and DeviceController, which are extensions of Kubernetes controller enabling management and data synchronization between the cloud and edge devices, and Cloud Hub, which is a WebSocket server enabling the communication between the controller and the edge part. The edge part that should be deployed on edge devices includes the following components: Edge Hub, which is a WebSocket client connected to the Cloud Hub mentioned above, that provides the interface for synchronization of cloud-side resource updates and status changes in the edge between the two main parts of KubeEdge, Edged agent for pod management, MetaManager that is the message processor between Edged and Edge Hub, EventBus MQTT client which enables the interaction with MQTT servers, DeviceTwin that stores and synchronizes device status to the cloud, and ServiceBus that provides the interface to interact with HTTP servers.

The main feature that enabled the inclusion of edge nodes to the remote cloud using the KubeEdge platform is the bi-directional WebSocket connection between the edge site and the cloud part, which is the extension of the existing Kubernetes platform. Thus, the Kubernetes apiserver component within the cloud does not have to communicate with worker devices directly, but instead, the communication is done through the WebSocket connection between the edge device (Edge Hub) and the cloud part (Cloud Hub). Since the edge device initiates this WebSocket connection between the edge and the cloud part, KubeEdge enables support for the inclusion of devices in private networks. However, networking between Kubernetes cluster and edge applications can still be a bottleneck that has yet to be resolved as described in [75]. Since KubeEdge is based on Kubernetes, its scheduling policy is implemented identically because the scheduling part was not extended. Thus, adapting the scheduling algorithm in KubeEdge implies the customization of the kube-scheduler component, as it is achievable in the same manner with the Kubernetes platform.

#### 3.1.4. ioFog

The last orchestration tool considered in our evaluation is the Eclipse ioFog. It is another open-source platform that facilitates deploying, running, and networking distributed microservices within edge computing environments [13]. The Edge Computing Network (ECN) on which the ioFog operates comprises one or more nodes, each running the Agent daemon service. The agent manages microservices running on the same node, which are usually deployed as Docker containers. The second essential component is the ioFog Controller, which orchestrates Agents in the ioFog network. It can be executed on any device, including those running the Agent component. Although a single Controller component is sufficient for smaller ECNs, multiple Controllers are enabled to support use cases that require greater resiliency. However, it is mandatory to ensure that this device has a stable hostname or static IP address to be reachable to all other Agents, enabling them to establish a connection with the Controller and pull the necessary control messages.

**Figure 5:**
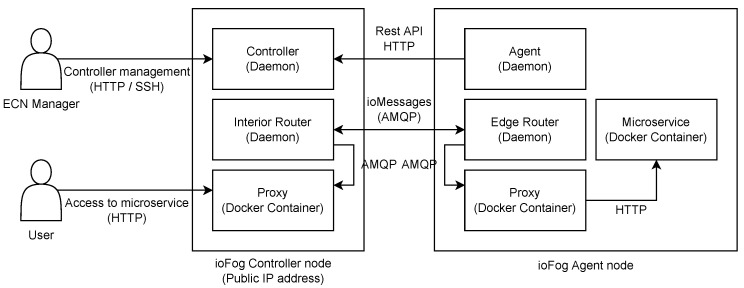
ioFog (v2) architecture.

The most recent version of ioFog (v2) has additional components in its architecture (depicted in Figure 5) compared to the previous version which are enrolled to enable communication between microservices and their exposure without direct access to the Agent [76]. An AMQP Router component enables communication among microservices and public port tunneling to these microservices. Each Controller and Agent component have their own Router component by default. An interior dispatch Router is deployed next to the Controller component first, and afterwards, each Agent component runs its own edge dispatch Router by default that is connected to the Controller’s interior Router. However, Routers can be modified by editing their *AgentConfig* to set up different network topologies and enable, e.g., direct communication between Agents on the same network without going back upstream to the default interior Router. The next important additional component is the Proxy that translates HTTP requests to AMQP messages when necessary. The Proxy is an additional container that boots up on the node where the service will be accessed by users (next to the interior Router) and also on the node where the targeted microservice is running (next to the edge Router).

The option of setting up an ECN that interacts with a Kubernetes cluster is also enabled by two additional components: Operator and Port Manager. The Operator is an internal ioFog component that manages ioFog control plane Custom resource, while Port Manager deploys proxies on the cluster necessary to expose microservices on external public ports. Setting up ioFog on Kubernetes requires that the Operator component is deployed first in the namespace so it could deploy an ECN in the same Kubernetes namespace when the control plane Custom resource is created. However, it is important to note that only the control plane can be deployed in Kubernetes while ioFog Agents remain outside the cluster. Thus, ioFog cannot schedule services on cluster nodes since these cannot act as ioFog agents.

### 3.2. Customization of Scheduling Algorithms

After architectural analysis of the chosen orchestration tools, in this section, we evaluate their potential to offer customization of their scheduling algorithms in order to provide better support for custom IoT use cases. In the context of edge computing, service orchestration implies service management and deployment across the available distributed execution environment. Each aforementioned orchestration tool offers the opportunity to set up a distributed cluster and then deploy services in it, but the logic of service scheduling and deployment differs. As K3s and KubeEdge are built upon Kubernetes, they utilize the same scheduling procedure as Kubernetes by default, so in this context, we will not consider them separately.

The Kube-Scheduler is a dedicated control plane component of Kubernetes that executes service scheduling in a two-step procedure [77]. When a pod is created, Kube-scheduler first executes the node filtering step, where it filters out all available nodes capable of running the specific pod based on its resource requirements, including primarily CPU and memory, but also some other parameters if necessary. The remaining nodes then enter into the second step of the Kubernetes scheduling procedure named scoring, where the goal is to determine the node that is the best fit to run a specific pod [78].

Each of these steps in the scheduling procedure can be customized by specifying various scheduling plugins that are parameters defining the custom criteria to achieve the desired scheduling execution logic. There are multiple options to specify preferences of the node selection process but most of them are based on the information characteristic for deploying applications within a cloud environment (e.g., specifying pod’s resource requirements, pod and volume collocation, etc.). However, customization based on the specific criteria is also possible by utilizing *nodeSelector*, *affinity/anti-affinity*, and *nodeAffinity* constraints. These mechanisms enable pod labeling, which guides the scheduling process towards the available nodes that meet the desired criteria. *NodeSelector* is the simplest form of node selection constraint that implies scheduling labeled pods on nodes with identical labels. Affinities provide similar functionality, but they allow defining more expressive types of constraints. Thus, they offer the opportunity to specify scheduling preferences for a pod without posing obligatory requirements that must be satisfied, so the pod can be scheduled even when the node with the given criteria is not found. The example of utilizing affinities to accomplish custom scheduling based on specific criteria is described in [77].

Thus, the conclusion is that Kubernetes does offer customization of its scheduling logic to a certain level, but it still has some deficiencies that are yet to be addressed in order for it to be utilized within the edge environments. These primarily imply its inability to specify custom variable QoS parameters that have to be taken into account while executing scheduling, and in addition to this, its inability to offer pod re-scheduling dependent on such QoS parameters. As QoS parameters are constantly changing within the edge environments (especially in terms of IoT use cases) and the benefits of the edge architecture are closely dependent on them, it is essential to efficiently resolve these shortcomings so that Kubernetes, and its edge-oriented distributions, become completely adapted for supporting its application within the edge environments. In its current state, custom parameters of available nodes and their assessment can be implemented in a separate service that can also be deployed within the Kubernetes cluster. Such a service could then update the metadata of the existing nodes with the latest values of custom parameters in a specific use case. However, such a service would also need information about the context of a service user in order to update the appropriate pod affinities so that the Kubernetes scheduler can redeploy the pod to the appropriate node when the scheduling procedure is triggered again.

IoFog is the only tool not built upon Kubernetes, so we examined its scheduling procedure separately. However, looking into its open-source resources ioFog also utilized a custom Kubernetes scheduler, but the component has not been maintained and upgraded in its newer versions. This may be due to its simplified logic, where the ECN manager is expected to trigger the service deployment with the command where a specific available worker node can be chosen. Therefore, the scheduling algorithm of ioFog schedules services in a similar manner as the default Kubernetes scheduler if the node is not specified within a command to deploy a service, or if it is, it deploys a service to the specific node. Customization of such a procedure implies implementing a separate service that would execute a complete overview of the execution environment and its specific QoS parameters. Upon this information, a separate scheduling service would then have to determine the most suitable node to deploy a service and trigger its deployment or redeployment over the ECN manager’s interface.

## 4. Performance Comparison of the Selected Container Orchestration Tools

The selected orchestration tools are compared in practice to test their applicability in the edge computing environment. For the comparison, we selected the container orchestration tools presented in the previous section, with the following latest stable releases at the time of conducting the experiments: *Kubernetes v1.26.1*, *K3s v1.26.1+k3s1*, *KubeEdge v1.12.1*, and *ioFog v2.0*.

Upon receiving the first request from a service client, the edge orchestration tool would ideally have to check if the service is already running at the nearest edge node to the service client and, if not, deploy or migrate the service to that particular node. In addition, further communication between the service and service user has to be executed directly, while the actions of the orchestration tool need to be triggered by the changes in the QoS for a particular client to fully exploit the benefits of edge computing. However, as described earlier in Section 3.2, none of the selected tools offer a solution to monitor and schedule services based on client QoS parameters. Therefore, for the comparison, we have only evaluated the possibility of connecting to the controller API and measured the time to start and migrate a particular service on the selected edge node.

### 4.1. Prerequisites

Before conducting the evaluation of the container orchestration tools, we needed to: (i) configure the evaluation environment and (ii) implement an edge service to be used in the evaluation.

#### 4.1.1. Environment Setup

**Figure 6:**
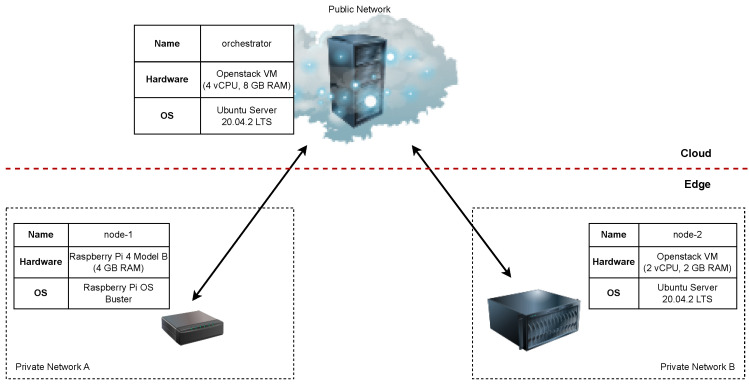
Configuration of the evaluation environment.

Figure 6 shows how the environment was configured to evaluate the orchestration tools with hardware and software details attached. Three nodes were used for the evaluation: one to run the ECC controller and two to serve as edge nodes. The edge node hardware was selected based on the survey results described in Section 2. The environment setup is built to answer the following questions:

- How does the orchestration tool handle edge nodes in private networks?
  - Edge nodes were placed in different private networks, and the controller was placed in the public network.
- How does the orchestration tool support node heterogeneity?
  - Nodes were deployed on both x86 and ARM processor architectures.
- How does the orchestration tool run on a resource-constrained edge node?
  - Node *node-1* runs on a single-board Raspberry Pi 4 computer.

It is important to note that all evaluations were performed in the same environment, and full cleanup occurred between subsequent evaluations.

#### 4.1.2. Edge Service Implementation

To conduct our evaluation, we first needed to design and implement a simple use case that would allow us to test the performance of selected orchestration tools and ultimately provide an answer to the aforementioned questions about their readiness for use in edge environments. The basic idea of edge computing is to perform data processing closer to data sources, which are often mobile, especially for IoT use cases. Therefore, our experiment uses a simple application that can be migrated depending on the location of the data source so that it can always be placed at the closest available edge processing node to the source. However, this goal must be achieved automatically by the orchestration tool, which must overcome all the previously described obstacles characteristic of edge execution environments.

The first step to building our experiment was to create a simple application and containerize it to enable its portability across available processing nodes. We implemented a Node.js HTTP server that returns a response to the GET request on the root path. The server was packaged as a Docker image and pushed to the Docker Hub [79] public container image registry.

### 4.2. Tool Evaluation

Once the environment was set up, we could evaluate the orchestration tools. The evaluation was performed in two parts: (i) cluster deployment evaluation and (ii) performance evaluation.

#### 4.2.1. Cluster Deployment Evaluation

The first part of the evaluation was to evaluate the deployment of the cluster using the selected tool. This evaluation step was intended to provide us with information about the complexity of the deployment, the support for different node platforms, and the memory footprint of the selected tool on the edge node. The deployment of the cluster was done in the following steps for each tool:

1. Reading and evaluating the tool documentation, focusing on cluster deployment instructions.
2. Installing and setting up the prerequisites for cluster deployment.
3. Deploying the cluster with the controller running on the cloud node *controller*.
4. Adding worker node *node-1* to the cluster and measuring its RAM consumption. Removing the node after the measurement and repeating this step several times to obtain an average value.
5. Adding worker node *node-2*.
6. Deploying our edge service on both nodes and connecting to it to verify that the cluster is working.

Table 3 shows the results of comparing selected container orchestration tools based on cluster deployment. Documentation quality and deployment complexity were closely related, as documentation was evaluated against cluster deployment instructions. Kubernetes is the expected winner regarding documentation quality, as it has the largest community and is an industry standard for container orchestration. On the other hand, the ioFog documentation lacked information about supported OS releases, which led to problems when deploying controller and worker nodes. Deploying the Kubernetes cluster was the most time-consuming of all the selected tools due to the fact that it is the only tool that does not provide built-in support for nodes in different subnets. Therefore, before deploying the Kubernetes cluster, we had to set up a VPN network, for which we used Wireguard [80]. Deploying KubeEdge requires deploying the Kubernetes control-plane node, which adds a bit of complexity, while ioFog requires setting up an SSH connection to each node in the cluster with *sudo* permissions. K3s was by far the easiest to deploy, requiring only one command to provision the controller and one command to join a node to the cluster. All selected tools supported nodes and containers running on both x86 and ARM processor architectures. None of the tools experienced significant performance degradation when running on an SBC, in this case, a Raspberry Pi 4.

As noted in Section 2, most authors consider using SBCs as edge nodes because they consume less power while being able to run high-performance tasks. Therefore, we decided to measure memory consumption on the node *node-1* running on the Raspberry Pi 4. This comparison gave unexpected results, showing that Kubernetes is undeservedly considered “*too heavy*” to run at the edge. The overhead of running the Kubernetes worker node on a Raspberry Pi is approximately the same as running a K3s or KubeEdge worker node, while K3s is known as “*lightweight Kubernetes*” and KubeEdge is known as the distribution designed for the edge. The worst performing is ioFog, which has almost five times higher memory footprint compared to the other three tools.

#### 4.2.2. Performance Evaluation

Once the cluster was deployed, we were able to measure the time needed to deploy and migrate a particular service with a selected tool. The performance evaluation was performed for each tool using the following steps:

1. Analyzing the controller API. Three main functions had to be performed through this API: service deployment on a selected node, service migration to another node, and service deletion.
2. Implementing an external service in Node.js that connects to the controller’s API and implements custom scheduling.
3. Performing service startup time tests on node *node-1*. This step was repeated several times to obtain an average value:
  (a) Obtaining the start timestamp.
  (b) Sending a request to deploy the service on node *node-1*.
  (c) Sending a request to the URL of the deployed service at regular intervals (every 100 ms) until a response is received.
  (d) Obtaining the end timestamp once the response is received and calculating the startup time.
  (e) Deleting the service.
4. Performing service migration time tests from *node-2* to *node-1*. This step was repeated several times to obtain an average value:
  (a) Sending a request to deploy the service on node *node-2*.
  (b) Sending a request to the URL of the deployed service at regular intervals (every 100 ms) until a response is received.
  (c) Obtaining the start timestamp once the response is received.
  (d) Sending a request to migrate the service to the node *node-1*.
  (e) Periodically (every 100 ms) sending a request to the newly deployed service URL until a response is received.
  (f) Obtaining the end timestamp once the response is received and calculating the migration time.
  (g) Deleting the service.

Table 4 shows the performance evaluation results by providing the average number of total service startup and migration times for each of the four container orchestration tools used in this study. The table also shows the overhead of a tool, which was determined by subtracting the container engine startup time from the total time. Since containerd [81] is a recommended container runtime for Kubernetes, we used it in this experiment for Kubernetes, K3s, and KubeEdge. Only ioFog services were deployed using the Docker Engine, as this is currently the only container runtime supported by ioFog. Therefore, before running our evaluation experiments, we tested the startup time of our service using these two container engines. The tests performed on *node-1* node that was used for comparison showed that containerd took 1.295 s and Docker Engine took 1.211 s to start the given service.

Note that ioFog is by far the worst performer in terms of service startup and migration time. We analyzed the agent logs and concluded that the message to start a particular container arrives about 30 s after the container start is triggered via the API, suggesting that the problem lies in the long request processing time at the controller. This is a known issue, identified in one of our previous works [16], that still needs to be resolved because it is the reason why ioFog is not yet suitable for running edge services on demand. The results show that K3s and KubeEdge have similar performance results, i.e., it takes about 2.8 s for the service to be deployed and available. Kubernetes, on the other hand, shows the best performance, with a startup time of approximately 1.8 s. This result was expected since both K3s and KubeEdge are Kubernetes *under the hood* with additional edge-specific components which may impact performance. KubeEdge introduces CloudCore and EdgeCore components between the Kubernetes API server and the container runtime on the node. In addition, the EdgeCore component includes a MetaManager that stores and retrieves metadata from the SQLite database installed on the node, which gives the node greater autonomy but also affects performance. On the other hand, K3s uses the lightweight SQLite [82] database for storage, which can cause performance degradation compared to Kubernetes with etcd [83]. Migration times are more or less comparable to the service startup time, meaning there is no significant overhead when performing migrations. This is important in edge environments with unstable nodes and networks where migrations occur with high frequency.

### 4.3. Discussion

In summary, Kubernetes performed excellently in both cluster deployment and performance evaluation. It has an unexpectedly low memory footprint on a resource-constrained edge node, so the only drawback to its use in the edge computing environment is the lack of support for nodes in private networks. Therefore, we had to set up a VPN network, which further complicates the setup and management of the cluster. Also, the utilization of VPN is not acceptable as a long-term solution to adopt Kubernetes within edge environments since the stability and performance would be highly dependent on the specific VPN solution. However, due to the large Kubernetes community, this orchestration platform frequently receives upgrades to enhance its performance and offer new features extending the scope of its usage. Received results of reduced memory footprint demonstrate its performance improvement in past years, and with other recent extensions that Kubernetes offers to enhance the support for its customization, such as e.g., *Operators* or *Scheduling Framework*, its utilization as the orchestration platform within edge computing setups is most likely to happen. Furthermore, recent development of *Scheduling Framework* offers a wider range of possibilities to adapt the scheduling policy of the cluster, which is also an important factor for the edge environment, as described in the text above. Thus, the only remaining challenge for using Kubernetes in edge environments is its support for built-in integration of nodes from private networks to the functional cluster environment.

K3s is specifically designed to overcome the drawback of setting up a VPN to enable the setup of Kubernetes cluster in the described test scenario, and evaluation results show that it has done so with minor performance degradation. K3s retains all the good Kubernetes practices, but contrary to its main slogan “*lightweight Kubernetes*”, it does not significantly reduce the memory footprint on an edge node. However, a memory footprint of 50 MB on a Raspberry Pi is still acceptable, so this is not really a drawback, just a misinterpretation. Using SQLite instead of the default etcd affects the footprint of the controller, but not the worker node. Another reason for the received degradation in results compared to Kubernetes is the communication between the api-server and the agent through the tunnel proxy. Thus, establishing a bidirectional link between the agent and the server offers the opportunity to enable communication towards nodes in private networks but at the cost of the additional overhead in communication, which reduces the considered performance score.

KubeEdge achieved similar evaluation results to K3s, with some minor improvements in memory footprint but with added deployment complexity, as the Kubernetes control plane must be deployed before KubeEdge can be deployed. It can be concluded that the inclusion of additional components in K3s and KubeEdge leads to a higher service startup time, which can still be improved, but makes Kubernetes distributions suitable for the implementation of edge solutions. Again, providing a bidirectional websocket connection between the cloud part and the edge resolves the challenge of including the edge nodes running in private networks to the cluster, but again with the cost of generating the overhead in communication that causes performance degradation. This points to the conclusion that the goal of including the edge nodes in the cluster is achievable, but then performance optimization is necessary to reach the results of the regular Kubernetes tool, or the communication will be slower, causing the degradation of overall migration performance.

Finally, ioFog has a single advantage of being deployable in relatively few steps, but its poor documentation, high memory footprint, and, most importantly, unacceptably high service startup time make it inadequate for edge orchestration. The current stable release of IoFog, *v2.0*, is already two years old, and since *v3.0* is still in beta, we hope that some of these major drawbacks will be fixed soon.

In general, received performance results from the described case-study scenario showcased that Kubernetes is the most promising solution for executing service orchestration within edge environments considering its best performance results, its active support, the size of the community and recent upgrades intended to enhance its extensibility and component customization. However, it still does not offer built-in support for including nodes running in private networks in the cluster environment. This requirement is resolved in other considered counterparts but with the cost of performance degradation that is more or less significant depending on the specific counterpart.

## 5. Conclusions and Future Work

In this paper, we first conducted a survey to provide an overview of the current state of the art in edge orchestration. In doing so, we focused on the question of whether orchestration tools have been used for experimentation in relevant work and, if not, what was the main reason for not using them. The survey results show that in most cases, the proposed edge orchestration algorithms or solutions have not been verified in a real-world environment using the available orchestration tools to schedule services according to user-defined QoS criteria. The customization of scheduling logic and tracking of various QoS parameters is done externally, which can lead to performance degradation in a real-world environment, as it is difficult to maintain an overview of the entire execution environment and all external parameters that can affect scheduling without orchestration tools.

Second, we evaluated the most commonly used container orchestration tools in literature (Kubernetes, K3s, KubeEdge, and ioFog) in a comparative experiment on a real edge deployment. The evaluation showed that Kubernetes performs very well even in a resource-constrained edge environment, disproving the assumption that only its distributions (K3s and KubeEdge) are more lightweight and suitable for edge computing nodes. However, K3s and KubeEdge solve the main problem of Kubernetes at the edge, namely by supporting nodes in private networks, albeit with some performance degradation. On the other hand, ioFog proved to be unsuitable for use in a dynamic edge environment due to its high service deployment time and a high memory footprint.

We can conclude that the potential exists for efficient scheduling within the edge computing environment by leveraging the available orchestration tools. However, there are still some challenges that should be addressed first to fully adapt these tools for use in distributed and unstable edge execution environments, as the tools were primarily designed for cloud environments.

Another important outcome of this study was to find out how custom scheduling can be integrated into existing container orchestration tools. In doing so, we found that the selected tools still do not provide the feature to specify custom and variable QoS parameters that need to be considered when running different scheduling algorithms. For this reason, they are not able to perform re-scheduling dependent on such dynamic QoS parameters. Therefore, in our future work, we intend to implement an external service and integrate it with Kubernetes (or one of its edge-oriented distributions) to continuously collect QoS information for each client-service connection so that re-scheduling can be performed when an important QoS parameter falls below a certain threshold. We also intend to design and implement a dynamic IoT case study to validate the selected container orchestration tool in an operational environment.

## Figures and Tables

**Table 1:** Related work search queries.

Query	Reference Count
(edge OR fog) AND orchestration	24
(edge OR fog) AND container	17
(edge OR fog) AND scheduling	11
(edge OR fog) AND serverless	7
(edge OR fog) AND placement	7
(edge OR fog) AND (kubernetes or kubeedge or k3s or iofog)	7
**Total number of relevant works**	**55**

**Table 3:** Cluster deployment comparison.

Parameter	Kubernetes	K3s	KubeEdge	ioFog
Documentation Quality	High	Medium	Medium	Low
Deployment Complexity	High	Low	Medium	Medium
Private Networks	No	Yes	Yes	Yes
Heterogeneity	Yes	Yes	Yes	Yes
Resource-Constrained	Yes	Yes	Yes	Yes
Memory Footprint (MB)	~50	~50	~40	~240

**Table 4:** Performance comparison results.

Time(s)	Kubernetes	K3s	KubeEdge	ioFog
Total Startup Time	1.799	2.798	2.858	34.537
Total Migration Time	1.838	2.782	2.781	23.244
Startup Time Overhead	0.504	1.502	1.562	33.326
Migration Time Overhead	0.542	1.486	1.485	22.032

## Data Availability

All data analyzed during this study are included in this article.
